# A Multi-Omics Study on the Effect of Helicobacter Pylori-Related Genes in the Tumor Immunity on Stomach Adenocarcinoma

**DOI:** 10.3389/fcimb.2022.880636

**Published:** 2022-05-10

**Authors:** Xinrui Wu, Aiwen Jian, Haidan Tang, Wangrui Liu, Fengyuan Liu, Shifan Liu, Huiqun Wu

**Affiliations:** ^1^ Department of Clinical Medicine, Medical School of Nantong University, Nantong, China; ^2^ School of Basic Medical Sciences, Shandong University, Jinan, China; ^3^ Affiliated Hospital of Youjiang Medical University for Nationalities, Baise, China; ^4^ Department of Interventional Oncology, Renji Hospital, Shanghai Jiao Tong University School of Medicine, Shanghai, China; ^5^ Department of Medical Imaging, Medical School of Nantong University, Nantong, China; ^6^ Department of Medical Informatics, Medical School of Nantong University, Nantong, China

**Keywords:** *Helicobacter pylori*, tumor immunity, mutation burden, immunotherapy, multiomics study

## Abstract

**Background:**

*Helicobacter pylori* (HP), a gram-negative spiral-shaped microaerophilic bacterium, colonizes the stomach of approximately 50% of the world’s population, which is considered a risk factor for gastritis, peptic ulcers, gastric cancer, and other malignancies. HP is also considered carcinogenic since it involves the mutation and damage of multiple HP-related genes. Stomach adenocarcinoma (STAD) is a common stom5ach cancer with a poor prognosis and high risk of metastasis in the advanced stage. Therefore, an early diagnosis and targeted therapies are needed to ensure a better prognosis. In this study, a scoring system was constructed based on three HP infection–related candidate genes to enable a more accurate prediction of tumor progression and metastasis and response to immunotherapies.

**Methods:**

HP infection–induced mutation patterns of STAD samples from six cohorts were comprehensively assessed based on 73 HP-related genes, which were then correlated with the immune cell–infiltrating characteristics of the tumor microenvironment (TME). The risk signature was constructed to quantify the influence of HP infection on individual tumors. Subsequently, an accurate nomogram was generated to improve the clinical applicability of the risk signature. We conducted immunohistochemical experiments and used the Affiliated Hospital of Youjiang Medical University for Nationalities (AHYMUN) cohort data set with survival information to further verify the clinical value of this risk signature.

**Results:**

Two distinct HP-related mutation patterns with different immune cell–infiltrating characteristics (ICIC) and survival possibility were identified. We demonstrated that the evaluation of HP infection–induced mutation patterns of tumor could assist the prediction of stages, phenotypes, stromal activity, genetic diversity, and patient prognosis. A low risk score involved an increased mutation burden and activation of immune responses, with a higher 5-year survival rate and enhanced response to anti-PD-1/L1 immunotherapy, while a high risk score involved stromal activation and poorer survival. The efficiency of the risk signature was further evidenced by the nomogram.

**Conclusions:**

STAD patients with a low risk score demonstrated significant therapeutic advantages and clinical benefits. HP infection–induced mutations play a nonnegligible role in STAD development. Quantifying the HP-related mutation patterns of individual tumors will contribute to phenotype classification, guide more effective targeted and personalized therapies, and enable more accurate predictions of metastasis and prognosis.

## Introduction

Stomach adenocarcinoma (STAD), the most common stomach cancer arising at (cardiac) or just below (noncardiac) the gastroesophageal junction, is among the most frequently observed and deadliest gastrointestinal malignancies, ranking fifth in incidence and third in mortality among all cancer types worldwide according to GLOBOCAN 2018 (Bray et al., 2018). Although the incidence and mortality of STAD have decreased since 1955, according to the World Health Organization, mainly due to a decrease in *Helicobacter pylorus* (HP) infections (Devesa et al., 1998), both remain high, with an estimated 1,220,662 incidences and 864,989 cases of associated mortality globally (Lunet et al., 2007; Karimi et al., 2014). The incidence and mortality of STAD vary among regions, ages, sexes, and races. Upon early diagnosis and timely treatment, the 5-year survival rate could peak at 97%; however, in cases of advanced-stage diagnosis or metastasis of STAD, the prognosis is considerably poorer with a 5-year survival rate of less than 30% (Allemani et al., 2015; Suzuki et al., 2016). Therefore, a better prognostic model is needed to predict STAD outcomes.

Infection is a key factor in the pathogenesis of STAD (Sukri et al., 2020; Kim and Wang, 2021). HP, a microaerophilic spiral-shaped gram-negative bacterium colonizing the human stomach, infects nearly half of the world’s population, making it the most prevalent human pathogen with relatively effective infectious strategies (Lina et al., 2014). The incidence of STAD in HP-positive patients is 3 to 6 times higher than that in negative patients (Kumar et al., 2020). However, its existence successfully escaped the attention of researchers until data were published by Barry Marshall and Robin Warren (Marshall and Warren, 1984). Since the discovery of HP, the understanding of the etiology of gastric diseases has advanced significantly. Although most HP-positive cases remain asymptomatic, it is commonly accepted that HP infection predisposes individuals to the initiation and development of a series of gastric diseases, including chronic active gastritis, peptic ulcer disease, duodenal ulcers, and even gastric adenocarcinoma in humans (Hagymasi and Tulassay, 2014; Shim et al., 2014; Malfertheiner et al., 2018). The discovery of HP confirmed the correlation between the etiology of gastric carcinoma and HP infection (El-Omar et al., 2003). Studies have shown that HP infection will lead to CpG island methylation of tumor-related genes in gastric mucosal epithelial cells (Baj et al., 2020; Kim et al., 2021; Wang et al., 2021). In addition, it may also cause cancer by inhibiting apoptosis (He et al., 2020; Yao et al., 2020). It is now a universally accepted carcinogen of gastric cancer, including diffuse-type STAD, and was listed as a class I carcinogen by the International Agency for Research on Cancer in 1994 (Correa et al., 2010). Three possible mechanisms of the initiation of HP-induced gastric cancer were proposed: epithelial cell DNA damage, repair process downregulation, mitochondrial DNA mutations, and transient mutator phenotype formation (Machado et al., 2010).

The tumor microenvironment (TME) of the solid STAD tumor consists of various cell types, including cancer cells, stromal cells, fibroblasts, smooth muscle cells, endothelial cells, and immune cells such as macrophages and lymphocytes. STAD is characterized by the immune cell infiltration of TME, mainly granulocytes, macrophages, and T lymphocytes (Caruso et al., 1999; Wu et al., 2012). It is universally acknowledged that the malignancy of STAD is influenced by the combination of environmental factors, intrinsic activities of cancer cells, and stromal and immune cells recruited and activated in the TME (Kim et al., 2016; Zeng et al., 2018). Mounting evidence has indicated the crucial role TME plays in the development, progression, prognosis, and prediction of the immunotherapeutic responses of STAD (Jiang et al., 2018). In addition, a correlation was found between host inflammatory response downregulation and parasite survival (Blaser, 1993; Wroblewski et al., 2009; Posselt et al., 2013). Primary host immune response mechanisms, including phagocytosis and natural killer (NK) cell activation, are downregulated by HP (Grebowska et al., 2008; Mnich et al., 2015; Rudnicka et al., 2015; Richter et al., 2016). Macrophage apoptosis and the inhibition of dendritic cell (DC) and macrophage maturation are also induced by HP infection and interrupt antigen presentation (Miszczyk et al., 2014). Altogether, the TME of STAD patients is significantly modified and characterized by HP infection to create an immunosuppressive and favorable microenvironment for its survival. Programmed death-ligand 1 (PD-L1) on gastric epithelial cells binds to the programmed cell death receptor 1 (PD-1) on the surface of T cells, promoting the activity of regulatory T cells that suppress the anti-tumor and anti-infectious responses. In the gastric tissue of STAD patients, the number of regulatory T cells is significantly increased, which was assumed to result from HP infection–induced PD-L1 expression according to some studies (Das et al., 2006; Enarsson et al., 2006; Beswick et al., 2007; Wu et al., 2010; Lina et al., 2015). Therefore, the utilization of immune-checkpoint blockade (ICB) therapy in HP-induced STAD patients is considered possible. In 2017, the use of pembrolizumab, an anti-PD-1 medication, in advanced or recurrent gastric cancer was approved by the Food and Drug Administration (Lina et al., 2015; Joshi et al., 2018). Despite encouraging results in a set of patients, the objective response rate remains relatively low, calling for the discovery of precise and accurate biomarkers and models to establish personalized ICB therapy. Therefore, here we aimed to classify the molecular phenotypes of STAD based on TME characteristics and construct a predictive risk signature for prognosis.

In this study, a comprehensive evaluation of the expression profiles of HP-related genes and the TME characteristics were obtained using R package. Based on the expression levels of HP-related genes, the STAD samples were first divided into two HPClusters. According to the differentially expressed genes (DEGs) identified in the two HPClusters, the samples were further classified into two distinct gene clusters. Subsequently, three core genes were identified using the least absolute shrinkage and selection operator (LASSO) and multivariate Cox regression analysis to construct a scoring system for predicting patient prognosis, survival, and responses to immunotherapy.

## Materials and Methods

### Stomach Adenocarcinoma Datasets Source and Preprocessing

STAD-related gene expression data and full clinical annotation with public access were searched in Gene Expression Omnibus (GEO; https://www.ncbi.nlm.nih.gov/geo/) and The Cancer Genome Atlas (TCGA; https://portal.gdc.cancer.gov/) database. Patients for whom survival information was lacking were omitted from further evaluation. Six online eligible STAD cohorts, including five GEO STAD cohorts (GSE15459, GSE62254, GSE84437, GSE38749, and GSE34942) and one TCGA-STAD, were selected in this study for further analyses. We excluded data lacking corresponding survival information and negative survival days. Additionally, information was also collected for a cohort from the Affiliated Hospital of Youjiang Medical University for Nationalities (AHYMUN). The AHYMUN (Guangxi, China) cohort included 104 patients diagnosed with STAD at the Department of Hepatology, Affiliated Hospital of Youjiang Medical University for Nationalities, between June 2009 and August 2018. Clinicopathological data were collected from pathology reports or electronic medical records. Samples of STAD and normal gastric tissues were collected during surgery and then processed and stored at the AHYMUN Tissue Bank before the experiments. The research scheme was approved by the research ethics committee of Youjiang Medical University. The raw CEL files were downloaded and a robust multiarray averaging method was adopted with the affy and simpleaffy packages to enable background adjustment and quantile normalization. For the TCGA datasets, RNA sequencing data (FPKM value) of gene expression were downloaded from the Genomic Data Commons (GDC; https://portal.gdc.cancer.gov/) and transformed into transcripts per kilobase million (TPM) values. Three datasets were merged and batch effects from non-biological technical biases were removed using the ComBat algorithm.

### Non-Negative Matrix Factorization Analysis of HP-Related Genes

A total of 73 HP-related genes were obtained from the GSEA-MSigDB website (http://www.gsea-msigdb.org/gsea/msigdb/index.jsp), where the keywords were *Helicobacter pylori* infection, including HP_HELICOBACTER_PYLORI_INFECTION and KEGG_EPITHELIAL_CELL_SIGNALING_IN_HELICO:ELICOBACTER_PYLORI_INFECTION. After HP infection, these 73 genes changed. Accordingly, we explored the possible potential biomarkers. The full details of these genes are shown in **Table S1**. Non-negative matrix factorization (NMF) analysis was employed to identify distinct HPClusters based on HP-related gene enrichment. R package was utilized to perform an NMF analysis of the HP-related genes. To explore the distinct characteristics in HP-related genes in biological processes, gene set variation analysis (GSVA) was conducted with the hallmark gene set (c2.cp.kegg.v7.2) derived from the MSigDB database. The clusterProfiler package was utilized to perform GO enrichment analysis for the HP-related genes to explore the enrichment features of these genes in different biological processes, cellular components, and molecular functions.

### Functional and Clinical Annotation of the HPClusters

The relationships between HPClusters and clinicopathological characteristics, recurrence-free survival, and prognosis were investigated to examine the clinical value of the two molecular subtypes. Characteristics such as age, sex, race, residual tumor, TNM stage, tumor stage, tumor grade, primary therapy outcome, and future status, were included in the analyses. The overall survival (OS) of the two HPClusters was assessed using Kaplan-Meier curves generated by the “survival” and “survminer” R packages. The clusterProfiler package was used to perform Gene Ontology (GO), Kyoto Encyclopedia of Genes and Genomes (KEGG) enrichment, and Disease Ontology (DO) analyses.

### DEG Identification and Functional Annotation

The empirical Bayesian approach of “limma” R package with a fold-change of 1.5 and an adjusted value of p < 0.05 was employed to identify the DEGs between the different HPClusters. A total of 706 DEGs were achieved (**Table S2**).

### Construction of the HP-Related Prognostic Risk Signature

A novel risk signature score was constructed to quantify the genetic characteristics of an individual tumor. To build the signature, the DEGs were identified from the HPClusters for LASSO analysis and then multivariate Cox regression analysis. Those DEGs determined as having a significant association with the STAD were selected. Second, patients with STAD were classified into distinct gene clusters using NMF analysis for further analyses. Finally, samples from the TCGA, GSE38749, and GSE84437 cohorts were randomly grouped into the training (n = 399) and testing (n = 399) sets at a ratio of 1:1 and the training set was used to construct the risk signature. Based on HP-related genes, the LASSO and Cox regression algorithm was used to minimize the risk of overfitting using the “glmnet” R package. The change trajectory of each independent variable was analyzed and a 10-fold cross-validation was utilized to construct the signature. Candidate genes were selected using multivariate Cox analysis for risk signature construction in the training set.

The risk signature was defined as follows: 


Risk score=Σ(Expi×Coefi)

where Expi and Coefi denote the expression and risk coefficient of each gene, respectively. According to the median score, patients in the training set, testing set, and all sets were divided into the low- and high-risk score groups. A Kaplan-Meier survival analysis was then conducted of each group and receiver operating characteristic (ROC) curves were generated.

### Somatic Mutation and PD-1/L1 Expression Analysis

CIBERSORT was employed to quantify the enrichment of infiltrating immune cells in the TME in the high- and low-risk score groups. The correlation between the immune cells and three selected genes was explored. Moreover, the association between the two groups and microsatellite instability (MSI) was also investigated. An Estimated STromal and Immune cells in MAlignant Tumour tissues using Expression data (ESTIMATE) score was also calculated to examine the purity of the tumor.

Subsequently, the tumor mutation burden (TMB) score was calculated for each STAD patient to investigate the somatic mutations between the high- and low-risk score groups. The differences in expression level of immune checkpoints between the high- and low-risk score groups were visualized using boxplots to predict the response to immunotherapy.

### Establishment and Validation of a Nomogram Scoring System

A predictive nomogram was developed using the “rms” package based on the independent prognosis analysis outcomes. In the nomogram scoring system, each variable was matched with a score, with the total score obtained by adding the scores across all variables of each sample. Next, ROC curves for sensitivity and specificity of distinct clinical characteristics and risk scores of 1-, 3-, and 5-year survival events were generated to identify the nomogram with the highest sensitivity.

### Verification of Signatures of Different Cohorts and Immunohistochemical Experiment

To test the stability of the signature, we substituted it into the GSE15459, GSE62254, GSE84437, GSE38749, GSE34942, and TCGA cohorts. The sample sizes of the GSE38749 and GSE34942 cohorts were too small (<30) so that the deviation would be too large. Therefore, the GSE84437, GSE38749, and GSE34942 cohorts were mixed for analysis. Furthermore, we searched PubMed for four signatures for STAD within a year (Shao et al., 2021; Wang and Liu, 2021; Zhang et al., 2021; Xiao et al., 2022) and compared them with our signature. Finally, immunohistochemical experiments were performed on the three genes in the obtained signature, and the survivals of the above three genes were verified *via* the AHYMUN cohort.

### Statistical Analysis

All statistical analyses were performed using R version 4.1.0. Statistics with p values < 0.05 were considered statistically significant.

## Results

**Figure 1:**
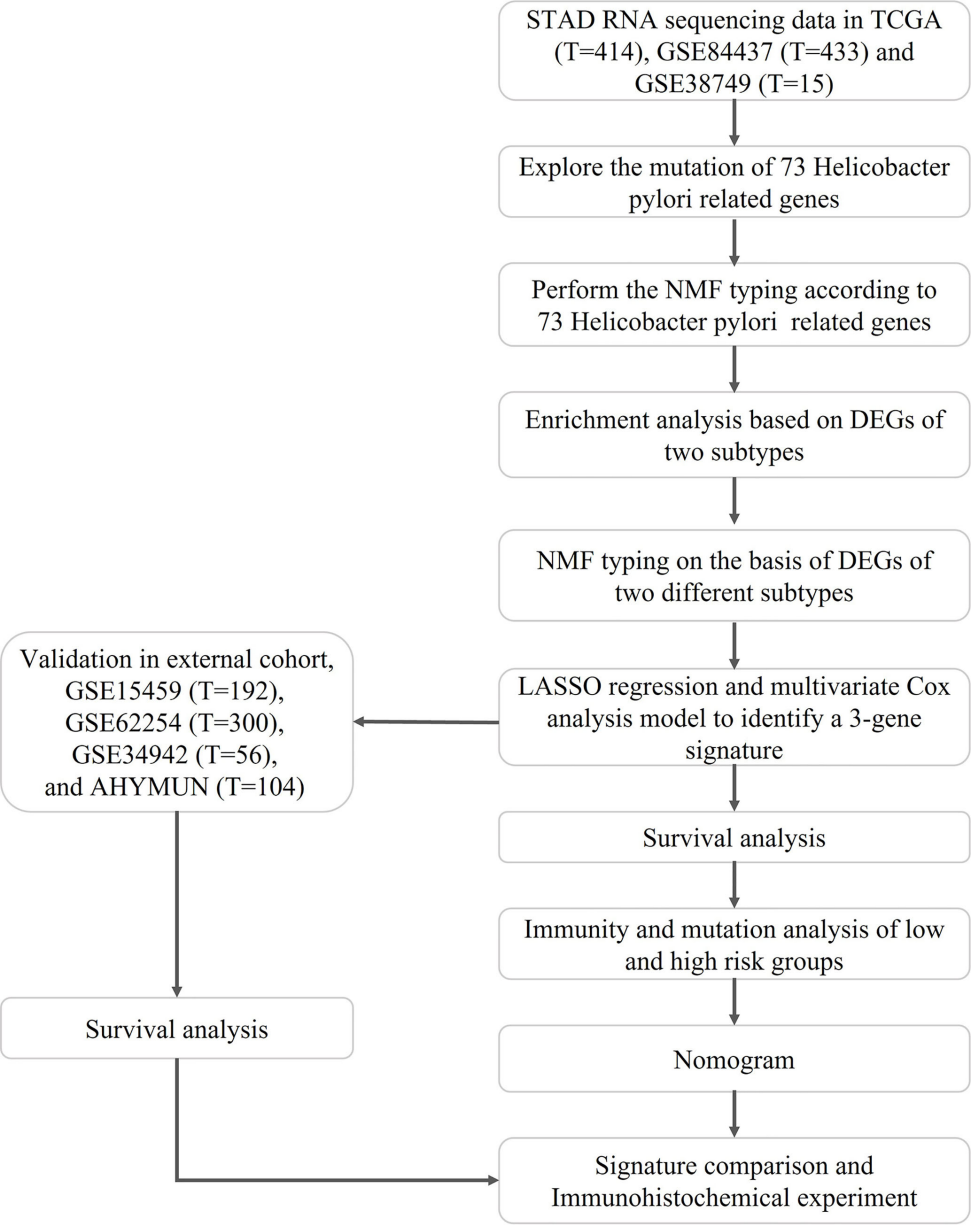
Flowchart of the construction and validation of HP related signature.

The flow of the study is shown in **Figure 1**, while the clinical characteristics of all STAD patients involved in the construction of the signature is shown in **Table 1**.

**Table 1:** Basic characteristics of STAD patients.

Characteristic	Levels	TCGA	GSE84437	GSE38749	p
n		414	433	15	
Gender, n (%)	FEMALE	145 (16.8%)	137 (15.9%)	6 (0.7%)	0.500
	MALE	269 (31.2%)	296 (34.3%)	9 (1%)	
Grade, n (%)	G1	10 (1.2%)	0 (0%)	0 (0%)	< 0.001
	G2	146 (16.9%)	0 (0%)	0 (0%)	
	G3	249 (28.9%)	0 (0%)	0 (0%)	
	unknow	9 (1%)	433 (50.2%)	15 (1.7%)	
Stage, n (%)	Stage I	52 (6%)	0 (0%)	0 (0%)	< 0.001
	Stage II	129 (15%)	0 (0%)	0 (0%)	
	Stage III	177 (20.5%)	0 (0%)	15 (1.7%)	
	Stage IV	40 (4.6%)	0 (0%)	0 (0%)	
	unknow	16 (1.9%)	433 (50.2%)	0 (0%)	
T, n (%)	T1	19 (2.2%)	11 (1.3%)	0 (0%)	< 0.001
	T2	87 (10.1%)	38 (4.4%)	0 (0%)	
	T3	190 (22%)	92 (10.7%)	0 (0%)	
	T4	113 (13.1%)	292 (33.9%)	0 (0%)	
	unknow	5 (0.6%)	0 (0%)	15 (1.7%)	
M, n (%)	M0	370 (42.9%)	0 (0%)	0 (0%)	< 0.001
	M1	27 (3.1%)	0 (0%)	0 (0%)	
	unknow	17 (2%)	433 (50.2%)	15 (1.7%)	
N, n (%)	N0	124 (14.4%)	80 (9.3%)	0 (0%)	< 0.001
	N1	113 (13.1%)	188 (21.8%)	0 (0%)	
	N2	81 (9.4%)	132 (15.3%)	0 (0%)	
	N3	85 (9.9%)	33 (3.8%)	0 (0%)	
	unknow	11 (1.3%)	0 (0%)	15 (1.7%)	
Race, n (%)	ASIAN	73 (8.5%)	0 (0%)	0 (0%)	< 0.001
	BLACK OR AFRICAN AMERICAN	10 (1.2%)	0 (0%)	0 (0%)	
	unknow	108 (12.5%)	433 (50.2%)	15 (1.7%)	
	WHITE	223 (25.9%)	0 (0%)	0 (0%)	
Tumor Status, n (%)	TUMOR FREE	208 (24.1%)	0 (0%)	0 (0%)	< 0.001
	unknow	85 (9.9%)	433 (50.2%)	15 (1.7%)	
	WITH TUMOR	121 (14%)	0 (0%)	0 (0%)	
Primary Therapy Outcome, n (%)	Complete Remission/Response	226 (26.2%)	0 (0%)	0 (0%)	< 0.001
	Partial Remission/Response	4 (0.5%)	0 (0%)	0 (0%)	
	Progressive Disease	65 (7.5%)	0 (0%)	0 (0%)	
	Stable Disease	16 (1.9%)	0 (0%)	0 (0%)	
	unknow	103 (11.9%)	433 (50.2%)	15 (1.7%)	
Residual_tumor, n (%)	R0	290 (33.6%)	0 (0%)	0 (0%)	< 0.001
	R1	15 (1.7%)	0 (0%)	0 (0%)	
	R2	15 (1.7%)	0 (0%)	0 (0%)	
	unknow	94 (10.9%)	433 (50.2%)	15 (1.7%)	
Age, meidan (IQR)		67 (58, 72.5)	62 (53, 68)	61 (52, 70)	< 0.001

### Landscape of HP-Related Genetic Alterations in STAD

**Figure 2:**
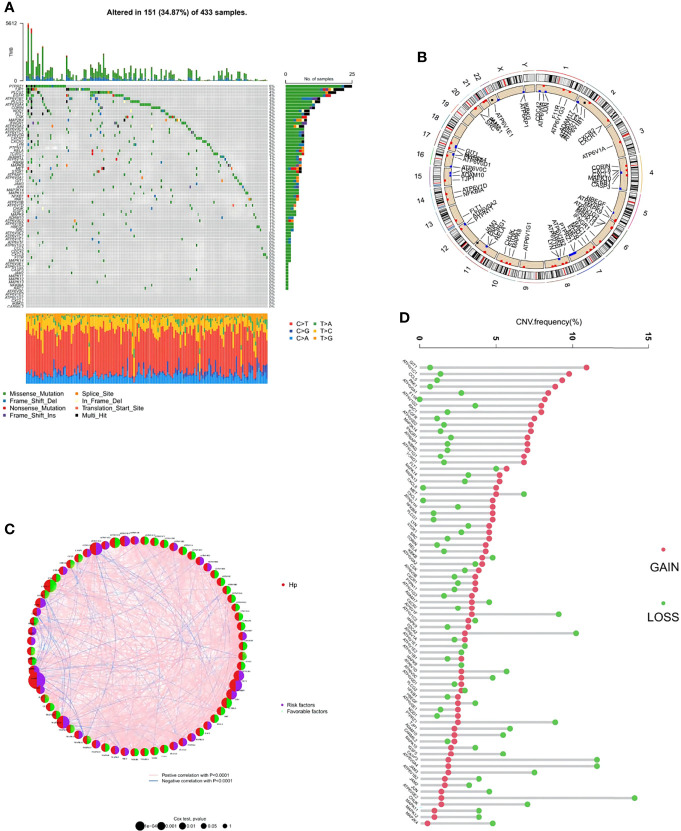
Genetic and transcriptional alterations of HP-related genes in stomach adenocarcinoma (STAD). **(A)** Mutation frequencies of 151 HP-related genes in 433 patients with STAD from the TCGA cohort. **(B)** Locations of CNV alterations in HP-related genes on 23 chromosomes. **(C)** The interaction between 73 HP-related genes in STAD. Red semicircles represent the gene is HP infection-associated. Purple and green semicircles represent risk factors and favorable factors, respectively. The circle size represented the effect of each regulator on the prognosis, and the range of values calculated by Log-rank test was p < 0.0001, p < 0.001, p < 0.01, p < 0.05 and P < 1, respectively. The line connecting the regulators represents their interaction, with the line thickness indicating the strength of the association between the regulators. Pink and blue stand for positive and negative correlation with statistical significance respectively. **(D)** Frequencies of CNV gain, loss, and non-CNV among HP-related genes.

Among the 73 HP-related genes included in this study, a summary analysis of somatic mutations in the TCGA-STAD cohort demonstrated a considerably high mutation incidence of HP-related genes under HP infection conditions (**Figure 2A**). In the TCGA cohort, 151 of 433 samples (34.87%) had noticeable HP-related gene alterations. Among the mutated genes, *PTPR71* had the highest mutation frequency, followed by *TJP1*, *PLCG1*, *EGFR*, *ATP6V1B1*, and *PLCG2*, while no alteration was observed in *CARMIL2*, *IKBKG*, *CXCL1*, or others.

Next, the copy number variations (CNV) in these HP-related genes were investigated, which indicated a prevalent alteration (**Figure 2B**). Among them, CNV increases were the most prominent in genes including *GIT1*, *ATP6V1C1*, *CCL5*, *PAK1*, and *ATP6V0A1*, while CNV decreases were the most significant in *ATP6V0E2*, *CASP3*, *ATP6V0A4*, *PTPRZ1*, and *CDC42*. The distributions of the CNV alterations in the 73 HP-related genes on human chromosomes are demonstrated in **Figure 2C**. To explore the interaction between the above genes and their roles in STAD development and progression, an HP-related gene interaction network was generated (**Figure 2D**). Most HP-related genes (n = 44) functioned as a favorable factor toward STAD, while *ATP6V0D2*, *ADAM10*, *FLT1*, and another 29 genes were considered the risk factors.

### NMF Clustering Based on the HP Genes in the STAD Samples

**Figure 3:**
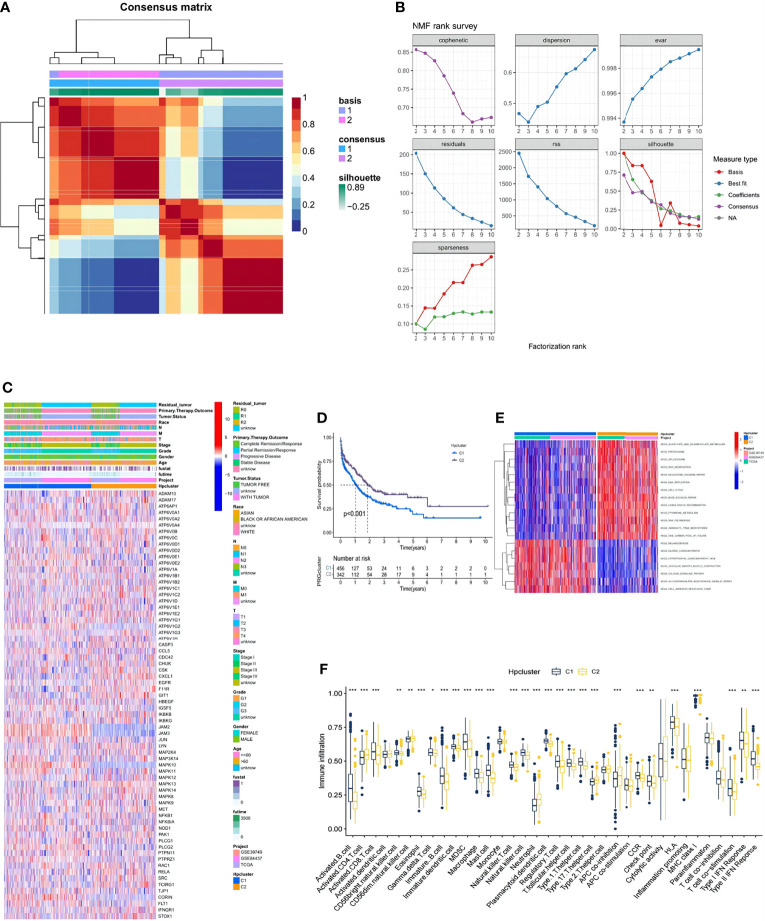
Clinicopathological and biological characteristics of two distinct HPclusters of samples from the combination of GSE38749, GSE84437, and TCGA-STAD cohorts, divided by NMF analysis. **(A)** NMF analysis heatmap defining two clusters (k = 2) and their correlation area. **(B)** NMF rank survey performed on the two HPclusters. **(C)** Clinicopathological annotation regarding the 73 HP-related genes. **(D)** Survival analysis of the two distinct clusters. **(E)** Heatmap of GSVA enrichment analysis. **(F)** Box plot demonstrating the immune cell-infiltrating characteristics of the two clusters. ns, no significance, p≥0.05; *, p< 0.05; **, p<0.01; ***, p<0.001.

In the combination of the GSE38749, GSE84437, and TCGA-STAD cohorts, an NMF analysis was conducted to classify the STAD samples into different molecular subtypes based on the expression of 73 HP-related genes. Two clusters were eventually identified, including 456 samples in the first subtype and 342 in the second (**Figures 3A, B**; **Table S2**). Unsupervised clustering of 73 HP-related genes in the combined cohort also revealed two distinct HP-related modification patterns with significant molecular and clinical characteristics (**Figure 3C**). We termed these two clusters HPClusters C1 and C2, respectively. A prognostic analysis of the two HPClusters indicated a significant survival advantage of the HPClusters C2 (**Figure 3D**). Markedly distinct KEGG pathway enrichment characteristics were also noticed between the two clusters in the GSVA enrichment analysis (**Figure 3E**). In addition, the two HPClusters also showed prominently distinct ICICs (**Figure 3F**). A prevalent amplification in immune cells, such as activated B cells, activated CD8 T cells, macrophages, and natural killer cells, was exhibited by the C1 cluster suggestive of inflammation-promoting characteristics, while activated CD4 T cells, neutrophils, and type 17 T helper cells were enriched in cluster C2. Interestingly, major histocompatibility complex class 1 (MHC-1) expression was significantly higher in the C2 cluster than in the C1 cluster, which is a possible explanation for the higher survival rate in the former than in the latter.

### GO, KEGG, and DO Enrichment Analyses of HPClusters

**Figure 4:**
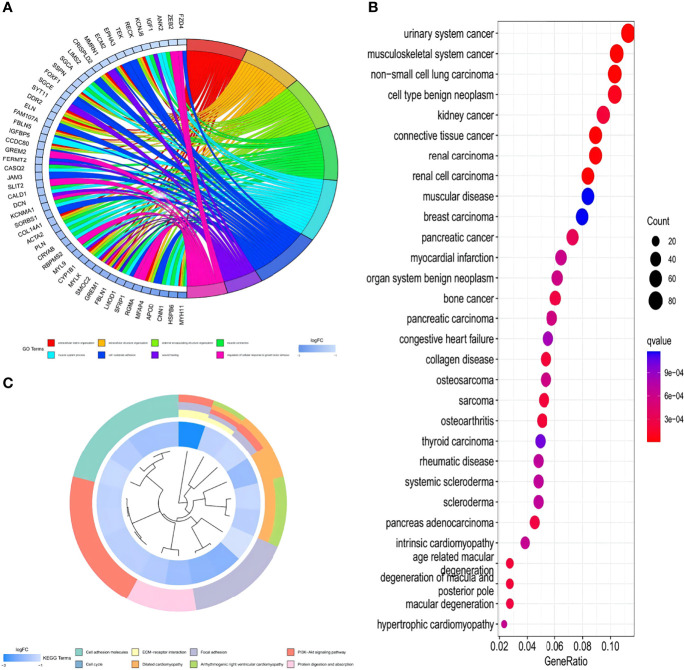
GO, KEGG, and DO enrichment analysis of the HP-related genes. **(A)** Enrichment circle graph of GO terms of the HP-related genes. **(B)** Bubble chart of Disease Ontology (DO) enrichment analysis of the HP-related genes. **(C)** Enrichment circle graph of KEGG biological process of the HP-related genes.

To further understand the characteristics and bio-behaviors of each HPCluster, the clusterProfiler package was employed to perform a GO enrichment analysis of the 73 HP-related genes (**Figure 4A**). These genes showed significant enrichment of biological processes related to cell–substrate adhesion and regulation of the cellular response to growth factor stimuli, which could partly explain the high malignancy and recurrence rates of STAD. Interestingly, these HP-related genes were also significantly enriched in the muscular system processes and muscle contractions. To explore the correlations between the HP-related genes and other diseases and malignancies, a DO analysis was performed, and the result showed that these genes were closely associated with musculoskeletal system cancer, consistent with the above findings (**Figure 4B**). Enrichment of the biological pathways was investigated using the KEGG analysis, demonstrating prominent amplification of pathways related to cell growth, cell proliferation, and angiogenesis (**Figure 4C**). The pathways related to multiple diseases, especially cardiomyopathy, were enriched in the C1 cluster, while those related to wound repair were amplified in the C2 cluster, corresponding with the previous findings.

### DEG Signatures in HPClusters and Functional Annotation

**Figure 5:**
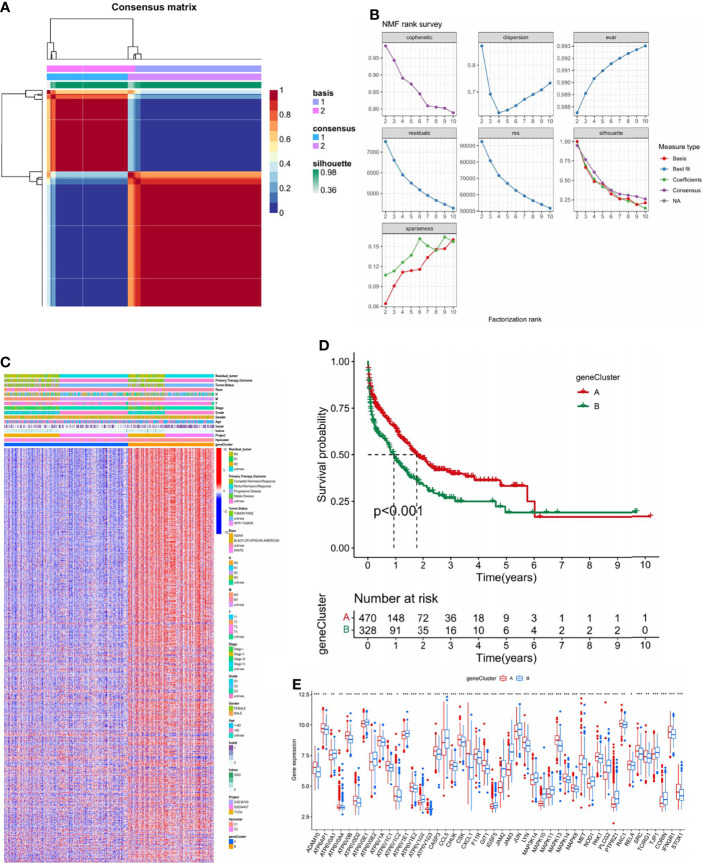
Identification of gene subtypes based on DEGs. **(A)** NMF analysis on the 706 DEGs. **(B)** NMF rank survey conducted on the DEGs. **(C)** Heatmap presenting the differentiation of the clinicopathological characteristics of the two gene clusters. **(D)** Survival analysis of the two gene clusters. **(E)** Gene expression analysis of the two gene clusters. ns, no significance, p≥0.05; *, p<0.05; **, p<0.01; ***, p<0.001.

To further investigate the genetic characteristics and potential biological function of each HPCluster, 706 DEGs were determined between the two HPClusters using the limma R package (**Table S3**). An NMF analysis was conducted on the 706 DEGs, and two completely distinct gene clusters were identified and termed A and B (**Figures 5A, B**; **Table S4**).

Considerably distinct clinical features are visible in the heatmap in **Figure 5C**. A significant survival advantage in gene cluster A was demonstrated in the subsequent survival analysis (**Figure 5D**). We compared the expression of HP-related genes in the two gene clusters (**Figure 5E**). Marked differences were found; most focused on higher expressions of favorable factors for STAD in gene cluster A, such as *ADAM10*, *ATP6V0B*, and *ATP6V1C1*, while the expression of risk factors such as *ATP6V0E1* and *ATP6V1E1* were higher in gene cluster B. This finding corresponds with the former discovery of a better prognosis in gene cluster A.

### Construction of the Risk Signature

**Figure 6:**
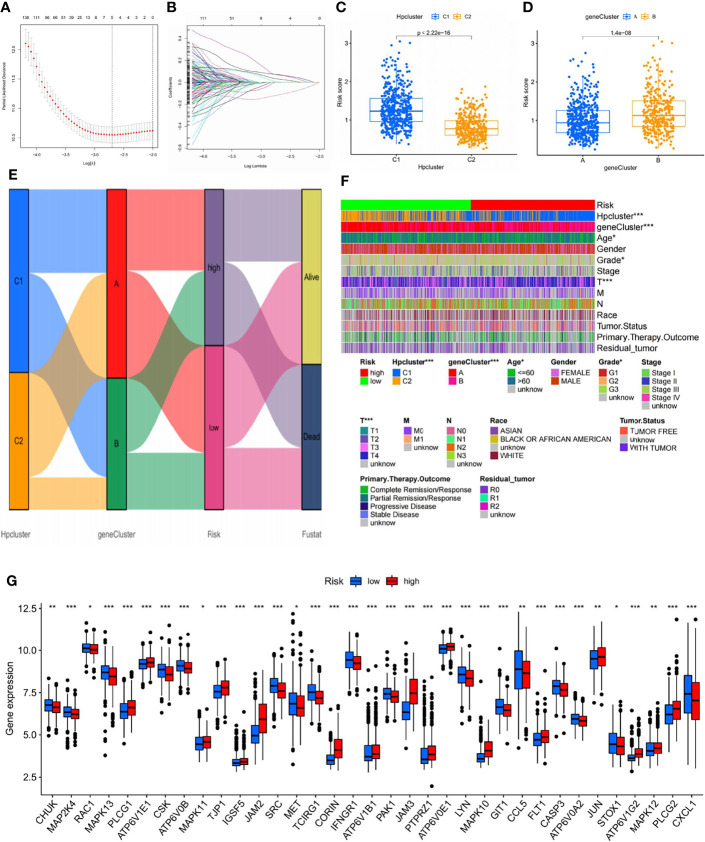
Selection of optimal prognostic signatures and constructure of risk signature in the training set. **(A)** Multivariate cox regression analysis for prognostic genes. **(B)** LASSO regression analysis for prognostic genes. **(C)** Differences in risk score among distinct HPclusters. **(D)** Differences in risk score among distinct gene clusters. **(E)** Alluvial diagram showing the changes of HPclusters, geneClusters, risk signature, and future status. **(F)** Heat map regarding the correlation between the risk signature, molecular, genetic classification, prognosis, and clinical features. **(G)** Differences in the expression of 39 DEGs among the low-risk group and high-risk groups. The blue bars and the red bars represent the groups with low and high m7Gscores, respectively. ns, no significance, p≥0.05; *, p<0.05; **, p<0.01; ***, p<0.001.

LASSO and multivariate Cox regression analyses were conducted and three core genes (*CRTAC1*, *BATF2*, and *CTHRC1*) were selected from the 706 DEGs to establish the risk signature (**Figures 6A, B**; **Table S5**). Caret R package was employed to randomly categorize the total STAD patients (n = 798) into two identical training (n = 399) and testing (n = 399) groups, in which the former was used to construct the signature.

According to the results of the multivariate Cox regression analysis, the risk score was constructed as follows:


Risk score=(0.1813×expression of CRTAC1)+(−0.2366×expression of BATF2)+(0.1800×CTHRC1).

A significant difference in risk scores was revealed between the distinct gene clusters (**Table S6**). Patients with a risk score lower than the average were categorized into the low-risk group (n = 177), while those with a risk score higher than the average were grouped into the high-risk group (n = 165). The scoring system was applied to all STAD samples, and patients classified into gene cluster B and HPCluster C1 had markedly higher risk scores than those in gene cluster A and HPCluster C2 (**Figures 6C, D**). An alluvial graph was generated to visualize the distribution of patients in HPClusters C1 and C2, gene clusters A and B, risk score, and future status, from which a higher survival possibility was noticed in the low-risk group (**Figure 6E**). To further investigate the relationships between risk score, molecular and genetic classification, prognosis, and clinical features, a heat map was generated for illustration (**Figure 6F**). This finding verified the above results that gene cluster A and HPCluster C2 had a better prognosis than gene cluster B and HPCluster C1. To better understand the relationship between the risk score and genetic behaviors, the expression of HP-related genes in the low- and high-risk groups were compared and visualized (**Figure 6G**).

**Figure 7:**
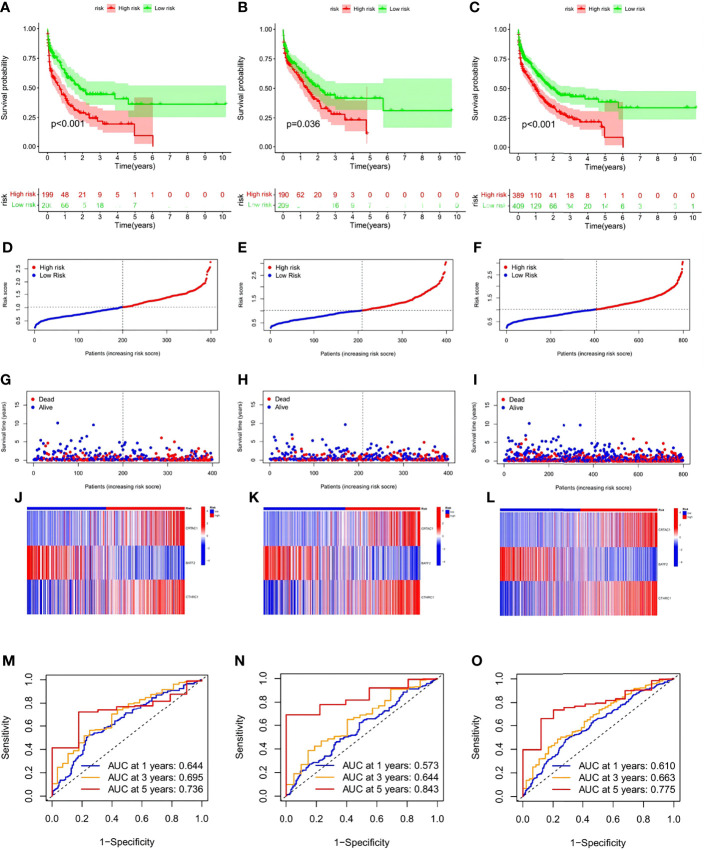
Validation of the risk signature in training, testing sets,and the two combined. **(A–C)** Kaplan-Meier survival analysis between the high- and low-risk score groups in training, testing sets, and the two combined. **(D–F)** Scatter plots showing the survival status of STAD patients with increasing risk scores. **(G–I)** Ranked dot plots indicating the risk score distribution. **(J–L)**. Heat maps showing the distribution of the three candidate genes. **(M–O)** ROC curves predicting the sensitivity and specificity of 1-, 3- and 5-year survival according to the risk signature.

To ensure convincing validation of the prognostic capability of the risk signature, a survival analysis was performed in the training and testing groups as well as the originally merged cohort and a similar result of survival advantages in the low-risk group were observed among analyses conducted in all sets (**Figures 7A–C**). In further analyses, favorable HP-related gene expression was higher in the low-risk group, while the opposite result was noted in the high-risk group. It was assumed that a lower risk score was associated with a higher survival possibility and that the survival rate would decrease as the risk score increased; this hypothesis was subsequently verified by the distribution plot of the risk score (**Figures 7D–I**). To assist in the comprehension of the influence of the three hub genes on oncogenesis, how the three core genes were distributed in the low- and high-risk groups within the training group, the testing group and total samples were illustrated *via* heatmaps (**Figures 7J–L**). To further verify the accuracy and efficiency of the risk signature, ROC curves in the training, testing, and combined groups were generated (**Figures 7M–O**). Analysis of the 1-, 3-, and 5-year prognostic categorization and prediction efficiencies demonstrated considerably higher AUC values of the risk signature, which substantiated the ability of the risk signature to predict the future status of STAD patients.

**Figure 8:**
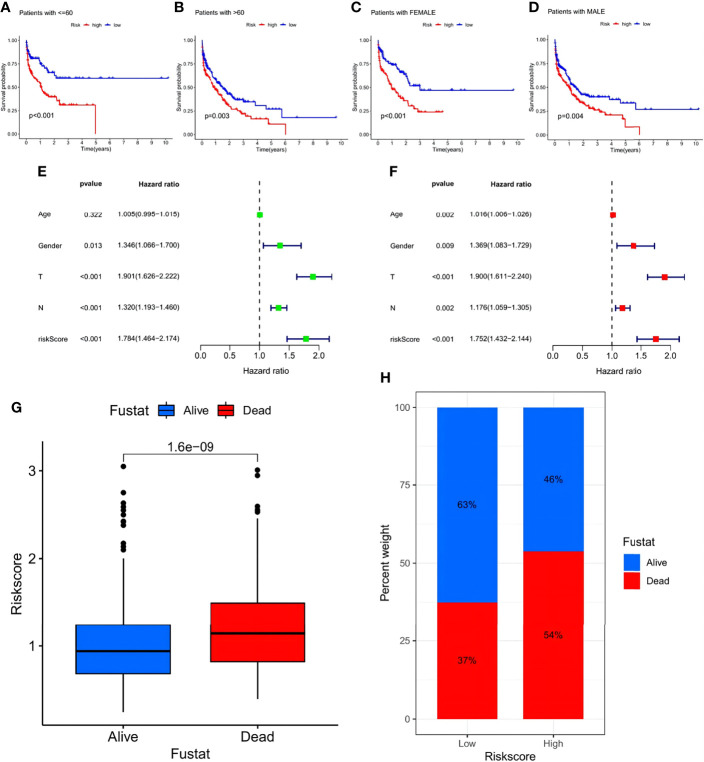
Investigation of the prognostic potential of the risk signature. **(A–D)** Survival analyses conducted on patients with distinct biological background, including age and gender. **(E)** Univariate COX regression analysis in the merged cohort. **(F)** Multivariate COX regression analysis in the merged cohort. **(G)** Evaluation of the risk score of samples with different future status. **(H)** Percentage of the alive and dead samples in low- and high-risk score groups, respectively.

In addition, a survival analysis was conducted in groups of different clinical features such as age (>60 years, <60 years) and sex with no results challenging the higher survival rate of the low-risk group. The above results suggest that lower risk scores were associated with higher survival possibilities (**Figures 8A–D**). Subsequent univariate and multivariate Cox regression analyses of the merged cohort also confirmed that the risk signature could act as an independent prognostic model for STAD (**Figures 8E, F**). To testify to the ability to predict future status, risk scores of STAD samples from alive versus dead patients were compared and visualized, and the former showed significantly lower risk scores (**Figure 8G**). Analyses of the future statuses of the low- and high-risk score groups revealed a prominently higher survival rate in the low- versus high-risk group (63% vs 46%, respectively) (**Figure 8H**).

### TME Characteristics and Potential Response to Immunotherapy

**Figure 9:**
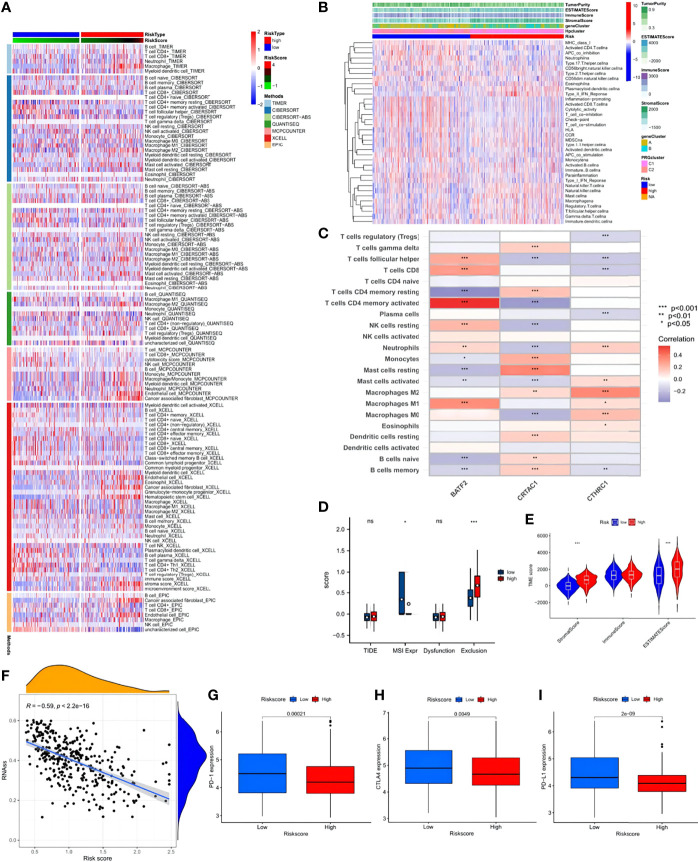
Immune annotation and correlation between HP infection and anti-PD-1/L1, anti-CTLA-4 immunotherapy. **(A)** Immune cell-infiltrating characteristics of high- and low-risk groups and their correlation with risk signature. **(B)** Relations between tumor purity, ESTIMATE score, immune score, stromal score, and different STAD phenotypes. **(C)** Correlation between the abundance of immune cells and three candidate genes. **(D)** Box plot suggesting the difference of response towards immunotherapy between high- and low-risk score group. **(E)** Violin plot illustrating the result of ESTIMATE analysis. **(F)** Relationships between the risk signature and cancer stem cell (CSC) index. **(G–I)** Expression of PD-1, CTLA-4, PD-L1 in low- and high-risk score groups, respectively. ns, no significance, p≥0.05; *, p< 0.05; **, p<0.01; ***, p<0.001.

CIBERSORT, Tumor Immune Estimation Resource, and other algorithms were employed to evaluate the association between risk score and immune cell infiltration, which was visualized *via* scatter diagrams and a heat map (**Figure 9A**). The analysis demonstrated that the risk score was positively correlated with macrophage M2, resting mast cells, monocytes, and resting memory CD4 T cells. Other immune cell types, such as M1 macrophages, resting NK cells, activated memory CD4 T cells, CD8 T cells, and follicular T helper cells showed negative correlations. However, no significant difference was seen between the low- and high-risk groups despite prominently lower stromal and ESTIMATE scores in the low-risk group (**Figures 9B, C**). Data of the STAD samples were analyzed further using the Tumor Immune Dysfunction and Exclusion algorithm. STAD patients in the low-risk score group showed higher MSI expression and significantly obvious T cell infiltration, indicative of better outcomes of ICB immunotherapy (**Figure 9D**). In the ESTIMATE analysis, significant differences were detected in stromal and ESTIMATE scores between the low- and high-risk score groups (**Figure 9E**). The high-risk score group exhibited prominently higher existence of tumor cells and higher tumor purity than the low-risk score group. In addition, the risk score and cancer stem cell (CSC) index values were synthesized to evaluate the correlation between the risk signature and CSC in STAD (**Figure 9F**). It was revealed that a negative linear correlation existed between the risk score and CSC index (R = -0.59, p < 2.2^-16^), indicating that the lower the risk score, the more distinct the stem cell properties and lower the cell differentiation degree. Subsequently, patients with lower risk scores showed obviously higher expressions of PD-1, PD-L1, and CTLA-4, suggesting a potential beneficial response in the low-risk score group to ICB therapy (**Figures 9G–I**).

**Figure 10:**
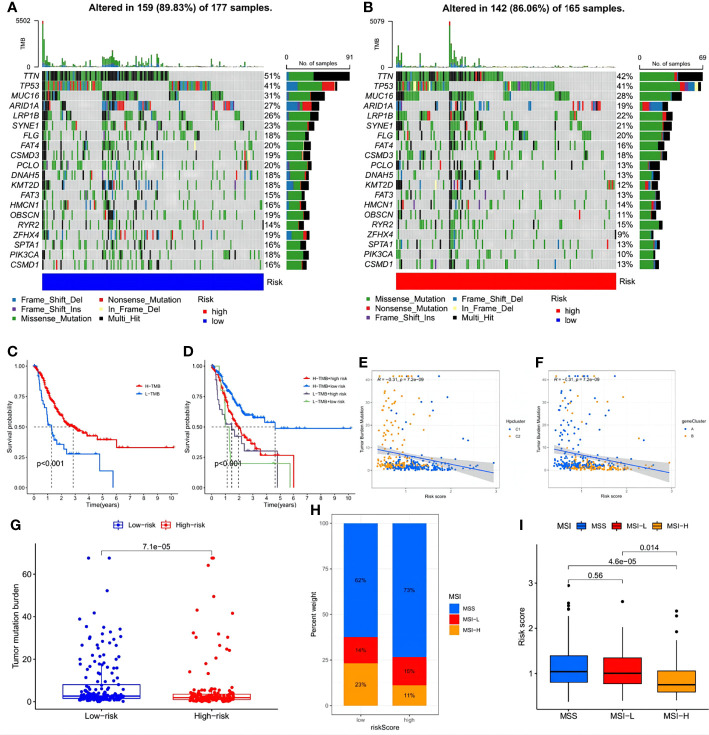
Exploration of the association between the tumor somatic mutation, MSI and risk signature. **(A, B)** The waterfall plots of tumor somatic mutation constructed by those with low- and high-risk scores, respectively. **(C)** Survival analysis on STAD samples with high and low TMB. **(D)** Survival analysis on STAD samples with different TMB and risk score. **(E, F)** Relationships between TMB and risk score based on HPclusters and gene clusters, respectively. **(G)** Distribution of STAD samples with low- and high-risk score in TMB. **(H, I)** Relationships between risk signature and MSI.

Further analysis investigated the relationship between immune infiltration and the three HP-related core genes. As **Figure 9E** shows, most immune cell types, especially activated memory CD4 T cells, showed a significantly positive correlation with *BATF2*, while the slight opposite occurred with *CRTAC1* and *CTHRC1*. The distribution differences of somatic mutation between the low- and high-risk score groups were then analyzed using the maftools package (**Figures 10A, B**). To further understand how TMB and risk score can predict the prognosis of STAD patients, more survival analyses were performed on groups with different TMB and risk scores. The low-risk score group with high TMB had the highest survival probability (**Figures 10C, D**). The correlations between TMB and risk score and between the HPClusters and gene clusters were then evaluated (**Figures 10E, F**). This result further verified that a low-risk score was linked to a high TMB. Clusters C2 and B also showed significantly higher TMB than the other two clusters. Consistent with the above findings, a more extensive tumor mutation burden was seen in the low-risk score group than in the high-risk score group, with overall mutation rates of 89.83% versus 86.06%, respectively, which was subsequently confirmed by the TMB quantification analyses (**Figure 10G**). To further assess the ability of the risk signature to predict STAD patients’ response to ICB therapy, we analyzed the MSI in the low- and high-risk score groups (**Figure 10H**). The low-risk score group contained a higher percentage of high MSI than the high-risk score group, suggesting that the former was more sensitive to immunotherapy and had greater clinical advantages. This correlation between the risk signature and MSI was confirmed further (**Figure 10I**).

### Nomogram Construction and Validation

**Figure 11:**
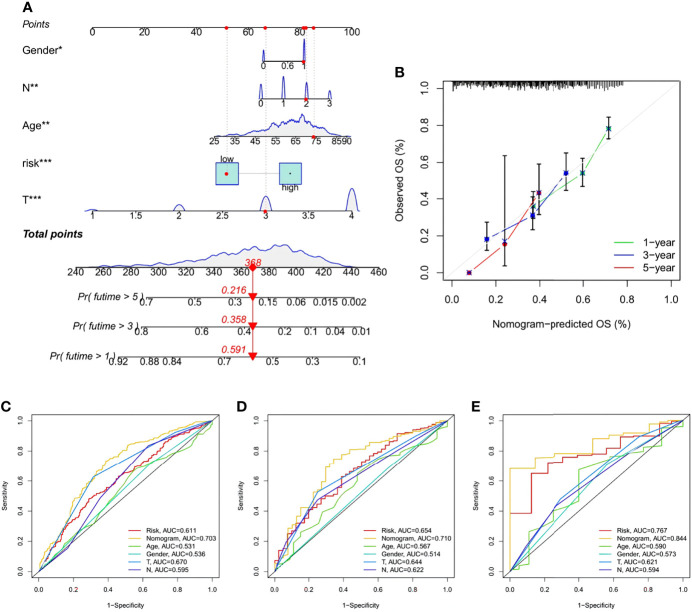
Construction and validation of a Nomogram. **(A)** Nomogram for prediction of 1-, 3-, and 5-year survival rate of STAD patients in the training set. **(B)** Calibration curves of the Nomogram for prediction of 1-,3-, and 5-year survival in the training set. **(C–E)** ROC curves for the sensitivity of different approaches in predicting the prognosis based on 1-, 3-, and 5-year survival rate of STAD patients in the training, testing, and all sets, respectively.

A nomogram containing the risk score and clinicopathological parameters was established to predict the 1-, 3-, and 5-year survival rates of STAD patients (**Figure 11A**). A subsequent calibration plot also suggested that the proposed nomogram had a similar performance compared to an ideal model (**Figure 11B**). To perform a more accurate and convincing validation, clinical ROC curves for 1-, 3-, and 5-year survival of STAD patients were generated and the nomogram showed the highest AUC values, suggesting its superior survival predictive ability compared to other parameters (**Figures 11C–E**).

### Test of the Signature *via* Online Database, AHYMUN Cohort, and Immunohistochemical Experiment

**Figure 12:**
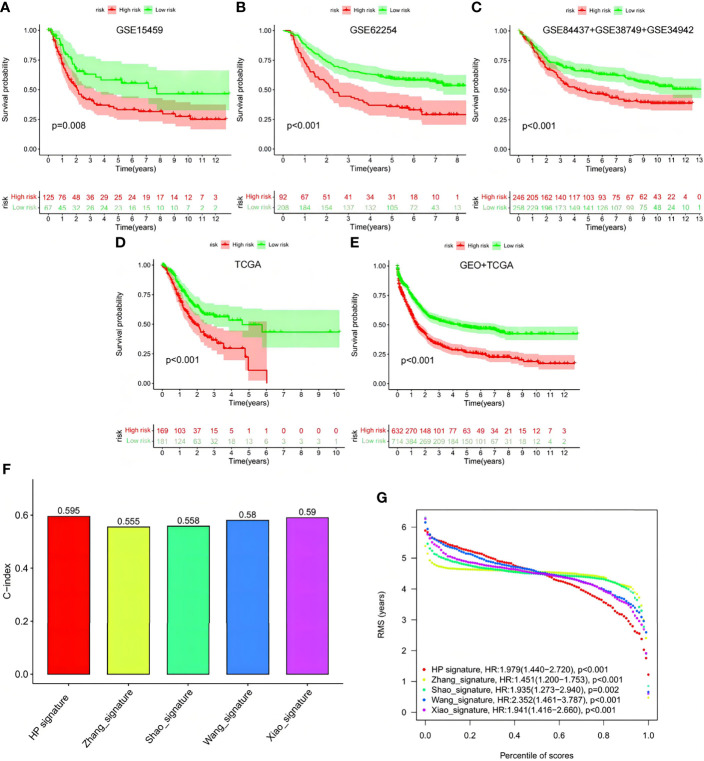
Verification of this signature by other ones and external cohorts. **(A–E)** Test of the signature *via* GSE15459, GSE62254, GSE84437+GSE38749+GSE34942, TCGA and GEO+TCGA. **(F, G)** Comparison among the signature with Zhang’s, Shao’s, Wang’s and Xiao’s ones.

To verify signature reliability, we applied the model formula to cohorts GSE15459, GSE62254, GSE84437+GSE38749+GSE34942, and TCGA-STAD (**Figures 12A–E**). We compared the results with the signatures reported by Zhang et al., Shao et al., Wang et al., and Xiao et al. (**Figures 12F, G**). Our signature had significant effects on the different online cohorts. Moreover, the C-index value of our signature was the highest among the five signatures, and its RMS value was <2, representing low dispersion and high reliability.

**Figure 13:**
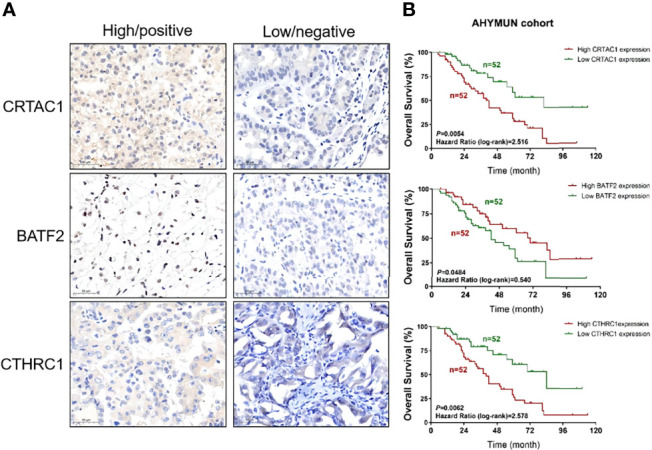
Test of the signature by experiment and AHYMUN cohort. **(A)** Immunohistochemical results of STAD. **(B)** Survival of CRTAC1, BATF2 and CTHRC1 with different expression levels in AHYMUN cohort.

The three genes in the signature, *CRTARC1*, *BATF2*, and *CTHRC1*, were analyzed by immunohistochemistry in the STAD samples (**Figure 13A**) and combined with the sample information of the 104 AHYMUN cohorts (**Figure 13B**). As previously analyzed, the high expression of *CRTARC1* and *CTHRC1* in the STAD samples represented lower OS, while higher *BATF2* expression predicted a better prognosis.

## Discussion

The present day sees a widespread interest in the pathogenesis and host–pathogen interaction in HP infection-related gastric carcinomas (Herrera and Parsonnet, 2009; Laird-Fick et al., 2016). Nevertheless, the majority still focus on the carcinogenic influence of virulence factors in the HP strain, such as *CagA*, *VacA*, *BabA*, and *oipA*, and environmental risk factors, including elevated pH of the gastric juice or the presence of nitrosamines and its precursors (Joossens et al., 1996; Gonzalez et al., 2006; Ofori et al., 2019; Sterbenc et al., 2019). On the molecular and genetic levels, despite general agreement on the direct causal relationship between the accumulation of mutation and carcinogenesis and proposed assumptions regarding HP infection–related carcinogenesis, no studies have elucidated the exact mechanism or the overall effect the infection had on the TME.

To compensate for the deficiency in this field, 73 HP-related genes were selected for analysis. These genes, according to the subsequent GO and KEGG analyses, were significantly associated with cell–substrate adhesion and the regulation of cellular response to growth factor; therefore, they were enriched in pathways related to cell growth, cell proliferation, and angiogenesis, mutations in which can significantly influence the regular biological behaviors of cells and thus lead to malignancies. Based on the expression of 73 HP-related genes and samples from two GEO cohorts and TCGA-STAD, two distinct HP-related gene mutation patterns were revealed with significantly different TME-infiltrating immune cell characteristics. Pattern C2 exhibited remarkable inflammation-promoting features. C2 was also characterized by prominent immune activation and higher MHC-1 expression. A distinction was also revealed in subsequent DO analysis, in which C1 presented a closer correlation with multiple diseases, especially cardiomyopathy, while C2 showed the amplification of pathways related to wound repair. Consistent with the above results, HPCluster C2 showed a significant survival advantage over HPCluster C1, which confirms the reliability of our classification of HP-related gene mutation phenotypes.

Since the TME ICICs have not yet been fully elucidated, the immune characteristics of different HP modulation phenotypes were thoroughly investigated. The subtypes were distinguishable by significant diversity in immune cell types, including activated B cells, activated CD8 T cells, eosinophils, T helper cells, mast cells, and macrophages, which were enriched in HPCluster C1, and activated CD4 T cells, neutrophils, APC co-inhibition, and MHC-1, which were amplified in HPCluster C2. In recent studies, T helper cells and mast cells were identified as valid prognostic immune cells associated with gastric cancer progression, which supports our findings (Wang et al., 2020). In subsequent analyses, the low-risk group with a better prognosis was identified with inflammation-promoting TME with a higher infiltration of activated CD8+ T cells, activated memory CD4+ T cells, and M1 macrophages, suggesting a critical role they played in STAD development. Amplification of native B cells and memory B cells in the high-risk score group was noticed. The enrichment of B cells, as suggested in previous studies, could be the strongest prognostic factor for prolonged survival and response to PD-1/L1 blockade in carcinoma (Petitprez et al., 2020). Therefore, despite the generally bad prognosis of the high-risk score group, B cell–targeted immunotherapy could be a possible approach to managing high-risk STAD.

Further, since the early detection and prediction of STAD recurrence are essential to a better prognosis, efficient biomarkers are desperately needed. However, the existing biomarkers, such as pepsinogen, gastrin, or HP serology combined with pepsinogen, are incapable of precisely presenting patient status, prognosis, or response to immunotherapy. Here, 706 DEGs were identified between the two HPClusters, upon which two gene clusters A and B were detected, with A showing higher expression of favorable factors and better prognosis and B the opposite. Among the 706 DEGs, three were selected for the construction of the risk signature. HPCluster C2 and gene cluster A showed lower risk scores than the other two. Significantly distinct clinicopathological features, prognosis, somatic mutation, TME characteristics, MSI, ESTIMATE score, and immunotherapy susceptibility were noted between the low- and high-risk score groups. On the whole, a low-risk score can be linked with a high survival rate and better outcome. This result affirms the vital role of the mutagenic effects of HP infection in gastric carcinogenesis by mechanisms previously suggested including the release of inflammatory byproducts, increase in reactive oxygen species, and accumulation of 8-hydroxydeoxyguanosine (Hahm et al., 1997; Farinati et al., 1998; Fu et al., 1999). The three candidate genes detected in this study were *CRTAC1*, *BATF2*, and *CTHRC1*. Evidence shows that cartilage acidic protein 1 (CRTAC1) is related to various malignancies, including bladder cancer and lung adenocarcinoma, by inhibiting the cell proliferation, migration, invasion, and epithelial–mesenchymal transition processes (Yang et al., 2021; Yu et al., 2021). However, the role of *CRTAC1* has not yet been reported in STAD or other gastric cancer. *BATF2* has been implicated in the progression and metastasis of diverse cancer in several studies (Dash et al., 2010; Wang et al., 2012). Of late, Xie et al. reported that N6-methyladenosine methylation modification–mediated *BATF2* acts as a tumor-suppressing factor in gastric cancer by inhibiting ERK signaling (Xie et al., 2020). The upregulation of *CTHRC1* is correlated with carcinogenesis, proliferation, invasion, and metastasis in numerous carcinomas, including gastric cancer (Ding et al., 2020; Mei et al., 2020). Consistent with the results of the above signature and AHYMUN cohort, a low-risk score represents higher *BATF2* expression and lower *CRTAC1* and *CTHRC1* expression, thus resulting in high survival.

In 2021, efforts were made to construct prognostic models with high predictive value using multiple aspects of the pathogenic mechanism of gastric cancer. Zhang et al. evaluated the prognostic value of mutation patterns related to survival in STAD and built a four gene–based risk score based on the mutation status of UTRN, MUC16, coiled-coil domain-containing protein 178, and HYDIN. Despite higher mutation counts among the low-risk group with a higher OS, whether this model can be applied to predict immunotherapy prognosis was not mentioned. Moreover, prognostic models established on the basis of immunologic and molecular subtyping can encompass this heterogeneity, thereby enabling a more accurate prediction of prognosis. Shao et al. focused on the emergence of pyroptosis in response to HP infection and built a scoring system for classification and prognosis prediction based on the expression levels of six pyroptosis-related genes (*GZMB*, *RBPMS2*, *CASP1*, *TAC1*, *TPM2*, and *GBP4*). However, this model did not deliver good predictive value for all-time survival stages under immunotherapy. A necroptosis-related prognostic signature based on four genes (*EZH2*, *PGAM5*, *TLR4*, and *TRAF2*) was established by Wang et al. according to the discovery of the association between necroptosis and antitumor immunity in immunotherapy. Nevertheless, no clinical information was involved in the model’s construction and verification. The latest prognostic model for STAD, a ferroptosis-related three-gene (*NOX4*, *CHAC1*, and *HIF1A*) prognostic signature, was developed by Xiao et al. Compared with the above four prognostic signatures, our model considered both heterogeneity and immune characteristics associated with HP infection in STAD and exhibited the highest accuracy and reliability among all existed models.

Through the integration of risk score and tumor stage, a quantitative nomogram was generated that further validated risk signature accuracy. Therefore, the risk signature can be used for prognosis stratification and classification of STAD patients, thereby assisting with the development of targeted and individualized immunotherapies.

## Conclusion

This work indicated how the mutation patterns induced by HP infection contributed to predicting patient prognosis and response to immunotherapy. The risk signature established in this study by quantification of the HP-related mutation patterns of STAD will contribute to phenotype classification, thereby guiding more effective targeted and personalized immunotherapies and enabling more accurate predictions of metastasis and prognosis.

## Data Availability Statement

The original contributions presented in the study are included in the article/**Supplementary Material**. Further inquiries can be directed to the corresponding author.

## Ethics Statement

The studies involving human participants were reviewed and approved by Youjiang Medical University. The patients/participants provided their written informed consent to participate in this study.

## Author Contributions

Conception and design: XW, AJ, HT, WL, and HW; Administrative support: XW and AJ; Provision of study materials or patients: HT and WL; Collection and assembly of data: XW, SL, and HW; Data analysis and interpretation: All authors; Manuscript writing: XW and AJ; Final approval of manuscript: All authors. XW, AJ, HT and WL contributes equally to this paper.

## Conflict of Interest

The authors declare that the research was conducted in the absence of any commercial or financial relationships that could be construed as a potential conflict of interest.

## Publisher’s Note

All claims expressed in this article are solely those of the authors and do not necessarily represent those of their affiliated organizations, or those of the publisher, the editors and the reviewers. Any product that may be evaluated in this article, or claim that may be made by its manufacturer, is not guaranteed or endorsed by the publisher.
